# Ravulizumab stabilizes life-threating intravascular hemolysis following delayed hemolytic transfusion reaction due to alloantibodies anti-e and anti-Jka: the first successful administration

**DOI:** 10.1007/s00277-025-06585-7

**Published:** 2025-08-28

**Authors:** Zoe Bezirgiannidou, Iliana Stamatiou, Theodoros M. Theodoridis, Eftychia Kontekaki, Emmanouil Panagiotopoulos, Christina Misidou, George Vrachiolias, Bouse Malkots, Menelaos Papoutselis, Ioannis Kotsianidis, Emmanouil Spanoudakis, Konstantinos Liapis

**Affiliations:** 1 Wireless Communications and Information Processing (WCIP) Group, Electrical & Computer Engineering Dept, Aristotle University of Thessaloniki, Thessaloniki, Greece; 2 Blood Transfusion, University Hospital of Alexandroupolis, Alexandroupolis, Greece; 3 Department of Hematology, University Hospital of Alexandroupolis Democritus University of Thrace Alexandroupolis, 681 00 Alexandroupolis, Greece

**Keywords:** Complement inhibitors, Ravulizumab, Delayed hemolytic transfusion reaction (DHTR), Hyperhemolytic syndrome, Alloantibodies, Intravascular hemolysis

## Abstract

This report describes the first successful administration of ravulizumab, a C5 complement inhibitor, in the treatment of life-threatening intravascular hemolysis (IVH) caused by delayed hemolytic transfusion reaction (DHTR) in a 22-year-old woman. The patient developed acute IVH with severe anemia and hemodynamic instability seven days after receiving a blood transfusion for posthemorrhagic anemia following a missed abortion. Laboratory investigations revealed anti-e and anti-Jka alloantibodies consistent with DHTR. Despite treatment, her hemoglobin level declined further, raising concerns for hyperhemolytic syndrome. After the administration of ravulizumab, her condition improved rapidly, and she was discharged with stable hemoglobin levels. Within three weeks there was full hematologic and biochemical recovery. This case demonstrates the therapeutic potential of ravulizumab in the management of severe complement-mediated hemolysis due to DHTR, and highlights the need for further research on complement inhibitors in similar conditions.

## Background

Delayed hemolytic transfusion reaction (DHTR) results from immunologic incompatibility between a transfusion recipient and the red blood cells (RBCs) of the blood donor. DHTRs are primarily mediated by a secondary anamnestic immune response triggered by alloimmunization against non-ABO antigens. Typically, the hemolytic event becomes evident one to two weeks post-transfusion [1]. Sensitization to red-cell antigens may result from previous transfusion, pregnancy, and less frequently hematopoietic stem-cell or solid organ transplantation [2].

In most cases, DHTR manifests as mild extravascular hemolysis mediated by macrophage clearance of transfused erythrocytes. Rarely, certain alloantibodies such as those against Kidd (Jka and Jkb) and P1 antigens, may induce complement activation on the membrane of transfused erythrocytes and severe intravascular hemolysis (IVH) [3].

Currently, there is no evidence-based study supporting the use of complement inhibitors in severe DHTR with IVH. Nonetheless, several case reports have demonstrated successful management of hyperhemolytic syndrome (HHS) with eculizumab [4–7]. Ravulizumab―a humanized monoclonal antibody against complement protein C5 that inhibits terminal complement activation―is approved for the treatment of patients with paroxysmal nocturnal hemoglobinuria (PNH) and atypical hemolytic uremic syndrome (aHUS). Its use in HHS and severe DHTR has not been previously described. We report, here, the first successful use of ravulizumab in a young woman with life-threatening IVH due to anti-e and anti-Jka alloantibodies.

## Case Report

A 22-year-old woman presented to our emergency department with fatigue, dizziness, back pain, vomiting, and reported passing dark, cola-colored urine. Seven days earlier, she had a missed abortion at week 6 managed with mifepristone and ergonovine maleate, which was complicated by severe vaginal hemorrhage requiring emergency surgical evacuation of the uterus. Due to blood loss, her hemoglobin decreased from 12.0 to 6.0 g/dL, and four units of packed red cells were transfused. Post-transfusion laboratory test results showed a hemoglobin level of 10.0 g/dL. She was prescribed oral iron and folic acid, and her hemoglobin increased to 11.1 g/dL (Table 1). The patient’s past medical history was notable for two first-trimester spontaneous abortions (<10 weeks of gestation). She had an uncomplicated full-term vaginal delivery two years ago.

 On presentation, she appeared lethargic and acutely ill. The initial vital signs revealed blood pressure 95/60 mmHg, pulse 120 beats per minute, respiratory rate 26 breaths per minute, temperature 36.5ºC, and pulse oximetry 99%. She had no lymphadenopathy or hepatosplenomegaly. Laboratory examinations showed a hemoglobin level of 9.2 g/dL (representing a 1.9 g/dL drop from the last measurement), a reticulocyte index of 1.69%, normal white-cell count and platelet count, markedly elevated lactate dehydrogenase level (2,298 U/L), undetectable haptoglobin (<5.83 mg/dL), and hemoglobinuria (3+) in the absence of visible red blood cells in the sediment (Table 1). 

 Clinical evaluation ruled out vaginal bleeding. Abdominal computed tomography excluded intraperitoneal hemorrhage. A hematology consultation was requested because of worsening anemia, and evaluation of the peripheral-blood smear revealed anisocytosis, basophilic stippling, spherocytosis, and rare schistocytes (<2 schistocytes per high-power field). The results of hemoglobin electrophoresis, a sickling test, glucose-6-phosphate dehydrogenase (G6PD) enzyme activity, a methyl-violet preparation for Heinz bodies, flow-cytometric immunophenotyping for paroxysmal nocturnal hemoglobinuria, and vitamin B12, folate, and ferritin levels were normal. Tests for rheumatoid factor, antinuclear antibodies, anti–double-stranded DNA antibodies, antiphospholipid antibodies, complement C3 and C4 levels, and antibodies to HBV, HCV, and HIV viruses were unremarkable.

 Immunohematological investigation using the ID-System (Bio-Rad Laboratories, CA, USA) revealed a weakly positive direct antiglobulin test (DAT) for anti-IgG (+/−) and a negative result for anti-C3c/C3d. The erythrocyte acid elution test (using DiaCidel reagents from Bio-Rad) was negative, and the cold agglutinin titer was not elevated. In contrast, the indirect antiglobulin test (IAT) was strongly positive (2+/3+). At least two alloantibodies (anti-e and anti-Jka) were identified, while there was a strong suspicion for additional unidentified alloantibodies. The patient’s phenotype was ccDEE [e(-)] and Jka(-), whereas both her husband and 2-year-old child had e(+), Jka(+) phenotypes. The 4 units of packed red cells that had been transfused seven days earlier had a ccDEe [e(+)] phenotype, two were Jka (+) and two were Jka (-). These units were cross-matched and transfused at a time when the patient's IAT was negative, and antigen-matched ccDEE units were unavailable in the hospital blood bank. On admission for hemolytic anemia, however, the patient's serum showed incompatibility with previously matched RBCs due to alloimmunization upon repeat crossmatch testing. In view of the history of recent transfusion, clinical picture, and the immunohematologic findings, a diagnosis of DHTR was made.

 Intravenous normal saline was administered, and she was treated with intravenous high-dose methylprednisolone, epoetin alfa 40,000 IU subcutaneously, and oral folic acid supplementation (10 mg daily). Despite this treatment, the patient became hypotensive (70/50 mmHg), and on repeat testing, her hemoglobin level had fallen to 6.3 g/dL (reflecting a 4.8 g/dL drop) (Table 1). The reticulocyte index was 1.89%. The progressive decline in hemoglobin levels despite treatment, along with the clinical deterioration, confirmed our suspicion of a life-threating complement-mediated immune hemolytic transfusion reaction. Although the patient’s hemoglobin levels did not drop below the pretransfusion nadir (6.0 g/dL), the overall drop of 4.8 g/dL exceeded the total hemoglobin increase obtained from the transfused units (4.0 g/dL), indicating rapid destruction of both transfused and autologous erythrocytes i.e. HHS.

 Given the high index of suspicion for HHS, the patient was managed according to the American Society of Hematology (ASH) 2020 guidelines for HHS, which suggest avoiding blood transfusions and initiating eculizumab if conventional therapy fails [8]. Eculizumab was not available in our hospital; however, ravulizumab—a long-acting complement C5 inhibitor—was available. Meningococcal vaccines and ciprofloxacin were given for prevention of meningococcal disease, and a single dose of ravulizumab (2700 mg) was administered intravenously over a two-hour infusion. Treatment with ravulizumab resulted in rapid clinical and hematological improvement. A striking feature in this case was that the dark brown discoloration of the urine resolved promptly during the administration of the drug.

**Fig. 1:**
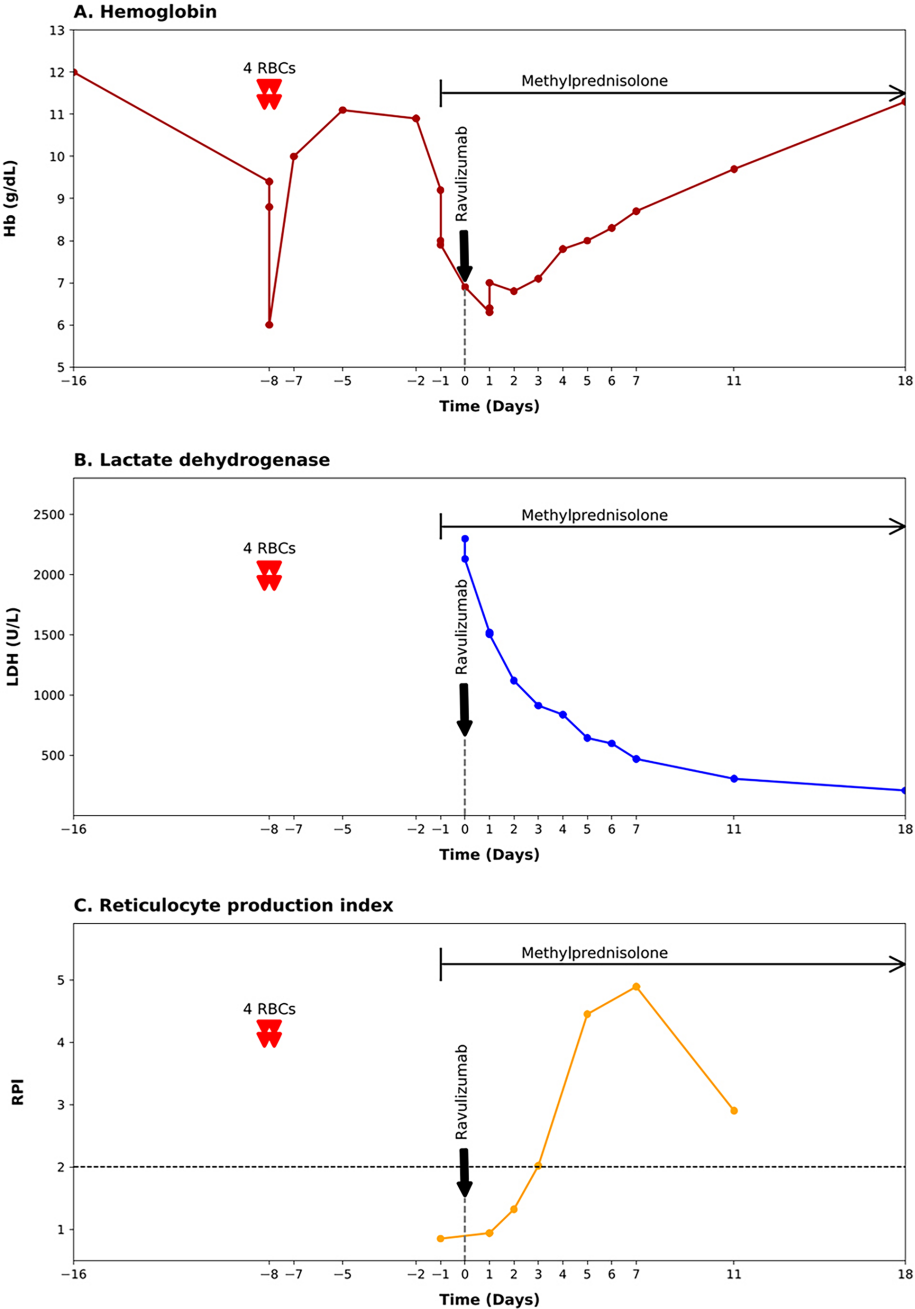
Time course of the patient’s hemoglobin (Hb), lactate dehydrogenase (LDH) levels, and reticulocyte production index (PRI). Also shown are therapeutic interventions (red-cell transfusions on day -8, ravulizumab infusion at time zero, and administration of methylprednisolone). A reticulocyte production index of ≥2 (dashed line) indicates an adequate bone-marrow reticulocyte response. 4 RBCs denotes transfusion of 4 red-cell units.

 On hospital day 8, the patient’s hemoglobin level increased to 8.7 g/dL, with a reticulocyte index of 7.34%. Intravenous iron supplementation (500 mg) was also given to support erythropoiesis. Normalization of hemoglobin and lactate dehydrogenase levels occurred by day 18 (Table 1). Methylprednisolone was tapered over two months. At a follow-up visit 8 months post-ravulizumab infusion, she was completely well. Alloantibodies were no longer detectable by IAT, even when more sensitive methods such as doubling both serum-to-erythrocyte ratio and incubation time were applied. The elution study and the cold agglutinin titer remained negative. The patient’s clinical course is illustrated in Figure 1

**Table 1:** Relevant laboratory values.

Test	Reference range	Day before treatment				Day of treatment					
		−8*	−5	−2	−1^†^	0^‡^	1	2	5	11	18
White-cell count (×109/L)	4.0–10.0	10.1	6.58	11.2	10.8	5.15	7.36	11.21	19.68	15.48	8.86
Hemoglobin (g/L)	12.0–16.0	6.0	11.1	10.9	9.2	6.3	6.4	6.8	8.0	9.7	11.3
Reticulocyte count (%)	0.5–2.5					3.07	4.27	5.46	11.56		
Platelet count (x109/L)	150–400	251	317	220	158	200	242	332	509	613	394
Partial-thromboplastin time (s)	25–35	27.3			21.7	33.5	29.6	27.5	25.1	25.4	
International normalized ratio (INR)	0.8–1.2	0.95			1.16	1.10	1.02	0.98	0.94	0.93	
Fibrinogen (mg/dL)	200–400				381	414	379	321	251	237	
Total Bilirubin (mg/dL)	0.3–1.2				3.73	1.29	0.45	0.28	0.28	0.28	0.32
Indirect Bilirubin (mg/dL)	0.2–0.8				3.06						
Alanine aminotransferase (U/L)	0–5	8		17	17	18	17	15	27	25	27
Aspartate aminotransferase (U/L)	0–35	12			81	50	22	11	10	10	11
Alkaline phosphatase (U/L)	30–120				39	42	38	41	36	51	53
Lactate dehydrogenase (U/L)	140–246					2298	1520	1119	643	304	207
Urea (mg/dL)	15–45	15		27	32	31	30	29	45	36	23
Creatinine (mg/dL)	0.6–1.1	0.51		0.64	0.67	0.60	0.57	0.59	0.51	0.55	0.58
C-reactive protein (mg/dL)	< 0.5				7.21	8.47	5.33	2.50	0.24		
Urine, hemoglobin					3+	3+			1+		0+
Urine, red cells per hpf					0–1	1–3			1–3		0–1

* Post hemorrhagic anemia (following surgical evacuation of the uterus) and transfusion of 4 units of packed red cells† Day of hospital admission for hemolytic anemia‡ Day of ravulizumab infusion

## Discussion

We present a case of a young woman who developed severe DHTR with IVH. HHS is a rare, life-threatening complication of DHTR characterized by complement-mediated destruction of both autologous and transfused RBCs. While this phenomenon is primarily reported in transfusion-dependent patients such as those with sickle cell disease (SCD) and thalassemia, it can also occur in transfusion recipients without underlying hemoglobinopathy, as described in the literature [11, 12]. The diagnostic criteria for HHS include: (a) a recent transfusion within the past 7 days (up to 21 days), (b) a rapid drop in the hemoglobin level of >25% below pretransfusion levels, (c) laboratory evidence of IVH, (d) reticulocytopenia (owing to inadequate marrow response), and (e) a decrease in HbA percentage in patients with hemoglobinopathy [8]. Our patient fulfilled most requirements for HHS. She had been transfused 7 days before admission. She experienced a hemoglobin decrease of 4.8 g/dL, while the total increase following the transfusion of 4 RBC units had been 4 g/dL, suggesting destruction of both donor and native erythrocytes. Although it was not possible to ascertain a >25% decline below baseline due to early intervention with complement inhibition, the clinical and laboratory features indicated ongoing rapid intravascular hemolysis. The presence of a low reticulocyte count (3%) and reticulocyte index (0.7%) during brisk intravascular hemolysis is compatible with HHS.

 Positive IAT or DAT are not essential for the diagnosis of HHS. Data indicates that only 67.4% of HHS cases have a positive IAT and only 45.7% have a positive DAT with IgG (40%), C3d (36%) or both (24%) detectable on the surface of RBCs [10]. In our case, IAT was positive with at least two alloantibodies (anti-e and anti-Jka), whereas DAT was only weakly positive for IgG (+/-). The absence of a reactive DAT for complement may be attributed to massive destruction of complement-coated RBCs or decreased sensitivity of the technical assays employed [8]. Complement activation after DHTR can lead to severe IVH. The theory of "bystander" hemolysis seems to be the most prevalent part of the immune dysregulation. Complement activation by autoantibodies or alloantibodies plays a key role in a subset of HHS cases. Both classical and alternative complement pathways are implicated in the hemolytic process. The assembly of the terminal C5b–9 membrane attack complex on the red-cell membrane leads to the rupture of red cells, which causes release of hemoglobin and RBC microvesicles into circulation that contribute to endothelial injury [13]. Additionally, cell-free heme and heme-loaded microvesicles may amplify complement activation (via activation of the alternative pathway) and cause systemic inflammation, leading to opsonization of RBCs by C3 and clearance of RBCs by macrophages via IL-6 activation. HHS is typically complicated by depressed erythropoiesis and reticulocytopenia, attributed to the systemic inflammatory response [8].

 In our patient, it could be hypothesized that initial sensitization occurred during the patient’s first pregnancy 2 years earlier, by exposure to fetal e(+) and Jka(+) antigens. Over time, alloantibody titers declined and were undetectable on routine pre-transfusion testing at the time of surgery for missed abortion that occurred in her last pregnancy. Quite possibly, the last pregnancy, due to its early gestational age (6 weeks), could not provide adequate antigenic stimulation to increase alloantibody titers. Transfusion with Jka(+) and e(+) RBCs, re-exposed the immune system to the relevant antigens, amplified the anamnestic response, increased alloantibody titers, and ultimately triggered hemolysis seven days later. Owing to suspicion for HHS, the patient was treated according to the ASH 2020 HHS guidelines, which recommend initial treatment with corticosteroids, intravenous immunoglobulin (IVIg), erythropoiesis-stimulating agents, and folate. The treatment protocol recommends against transfusions in order to avoid devastating hemolysis, but if transfusion is deemed necessary, RBCs must be matched more extensively (extended phenotype) for C/c, E/e, K, Jka/Jkb, Fya/Fyb, and S/s antigens [8, 9, 14]. In our patient, alloantibody screening identified two antibodies (anti-e and anti-Jka), while there was strong suspicion for more alloantibodies. Due to unavailability of fully matched units, transfusion was deemed unsafe. According to current guidelines [8], transfusion in HHS should be reserved for life-threatening situations or when Hb drops below the nadir of 3-3,5 gr/dl. In our patient, hemoglobin levels remained above 6.0 g/dL. We did not use IVIg because we suspected that our patient had complement-mediated acute intravascular hemolysis. IVIg acts mainly by saturating Fcγ receptors on macrophages, thereby preventing extravascular hemolysis. It has no direct inhibitory effect on complement activation or membrane attack complex (C5b–9) formation, and thus is not effective in rapid complement-mediated IVH.

Treatment of HHS involves suppression of the immune system (e.g. use of corticosteroids) and complement inhibition. Several cases of HHS in patients with SCD or thalassemia have been treated successfully with eculizumab. ASH guidelines recommend treatment with a C5 inhibitor (eculizumab) as a second-line therapy, if the patient does not respond to conventional treatment with high-dose corticosteroids and IVIg [9]. In accordance with these guidelines, we decided to treat this patient with a C5 inhibitor (ravulizumab), although she had no laboratory evidence of complement activation or consumption (serum levels of complement C3 and C4 were within normal limits and DAT was negative for C3c/C3d). Total CH50 level, the assay that measures the activity of the classic complement pathway, was not available in our hospital. Recently, sutimlimab, a C1s inhibitor, has been approved for the treatment of cold agglutinin disease (CAD) [15], but its use in hemolytic transfusion reactions is unknown. Additional supportive treatment options include inhibition of RBC clearance via macrophage inhibitors and stimulation of erythropoiesis with erythropoietin and administration of folate, vitamin B12, and intravenous iron [9].

 Eculizumab has been used in the management of HHS; however, we are unaware of other reports in the literature concerning the use of ravulizumab in patients with DHTR. Ravulizumab leads to rapid and efficient terminal complement blockade in PNH and aHUS [16–19]. In our case, ravulizumab was chosen due to its availability in our hospital, originally reserved for an upcoming scheduled dose of a patient with PNH one month later, allowing for its subsequent substitution. Ravulizumab was administered after approval from our Ethics Committee and discussion with the patient and her family. During the administration of ravulizumab, the patient had a dramatic clinical response with rapid clearance of her urine from dark brown to yellow, indicating immediate interruption of IVH. 

To our knowledge, this is the first case of a successful outcome following ravulizumab administration in severe DHTR. Our case highlights the efficacy of ravulizumab in managing life-threatening IVH and HHS and suggests that it has a role in the management of IVH caused by complement activation. Future clinical studies are needed to validate the efficacy of complement inhibitors in severe DHTR and HHS. Furthermore, the constant availability of complement inhibitors for urgent use―especially in hospitals dealing with severe hematologic disorders―is of utmost importance.

## Data Availability

Patient consent is provided within the supplementary information files.
